# Evidence of the adverse effects of air pollution on the population’s health in Spain: analysis of the economic costs of premature deaths

**DOI:** 10.1590/0102-311XEN145922

**Published:** 2023-08-11

**Authors:** Bruno Casal, Berta Rivera, Luis Currais

**Affiliations:** 1 Universidade da Coruña, A Coruña, España.

**Keywords:** Particulate Matter, Premature Mortality, Health Risk, Economic Burden, Material Particulado, Mortalidad Prematura, Riesgo a la Salud, Dificultades Económicas, Material Particulado, Mortalidade Prematura, Risco à Saúde Humana, Dificuldade Econômica

## Abstract

Exposure to ambient air pollution increases mortality and morbidity, leading disabilities, and premature deaths. Air pollution has been identified as a leading cause of global disease burden, especially in low- and middle-income countries in 2015 (*Global Burden of Diseases, Injuries and Risk Factors Study*, 2015). This study explores the relation between mortality rates and particulate matter (PM) concentrations in the 50 Spanish regions for the period 2002-2017. Moreover, we estimated the premature deaths due to PM in Spain according to welfare and production losses in 2017. Random-effects models were developed to evaluate the relation between mortality rates and PM concentrations. The economic cost of premature deaths was assessed using the Willingness to Pay approach to quantify welfare losses and the Human Capital method to estimate production losses. _PM10_ concentrations are positively related to mortality due to respiratory diseases and stroke. Based on 10,342 premature deaths in 2017, losses in welfare amount to EUR 36,227 million (3.1% of Spanish GDP). The economic value of current and future production losses reached EUR 229 million (0.02% of GDP). From a social perspective, air pollution is a public health concern that greatly impacts health and quality of life. Results highlight the need to implement or strengthen regulatory, fiscal, and health public policies to substantially benefit the population’s health by reducing their exposure to air pollution.

## Resumen

La exposición a la contaminación atmosférica aumenta la mortalidad y la morbilidad, lo que conduce a la discapacidad y a la muerte prematura. La contaminación del aire se identificó como una de las principales causas de la carga mundial de enfermedades, sobre todo en países de ingresos bajos y medianos en el 2015 (*Global Burden of Diseases, Injuries and Risk Factors Study*, 2015). Este artículo explora la relación entre las tasas de mortalidad y la concentración de material particulado (PM) en las 50 regiones españolas desde el 2002 hasta el 2017. Además, se realizó una estimación de las muertes prematuras provocadas por PM en España en términos de bienestar y pérdidas de producción en el 2017. Se desarrollaron modelos de efectos aleatorios para estudiar la relación entre las tasas de mortalidad y las concentraciones de PM. El costo económico de las muertes prematuras se evaluó usando el enfoque “disposición a pagar” para monetizar las pérdidas de bienestar y el método del capital humano para estimar las pérdidas de producción. Las concentraciones de PM_10_ están positivamente asociadas con la mortalidad por enfermedades respiratorias y accidente cerebrovascular. Con base en 10.342 muertes prematuras en el 2017, las pérdidas en el bienestar social ascendieron a EUR 36.227 millones (3,1% del PIB español). El valor económico de las pérdidas de producción presentes y futuras llegó a EUR 229 millones (0,02% del PIB). Desde un punto de vista social, la contaminación del aire es un problema de salud pública que tiene un gran impacto en la salud y en la calidad de vida. Los resultados ponen de manifiesto la necesidad de implementar o de fortalecer políticas públicas regulatorias, fiscales y de salud para obtener beneficios sustanciales para la salud con la reducción de la exposición.

## Resumo

A exposição à poluição do ar ambiente aumenta a mortalidade e a morbidade, levando a incapacidades e mortes prematuras. A poluição do ar foi identificada como uma das principais causas da carga global de doenças, principalmente em países de baixa e média renda em 2015 (*Global Burden of Diseases, Injuries and Risk Factors Study*, 2015). Este artigo explora a relação entre as taxas de mortalidade e a concentração de material particulado (PM) nas 50 regiões espanholas de 2002 a 2017. Além disso, foi realizada uma estimativa das mortes prematuras causadas por PM na Espanha em termos de bem-estar e perdas de produção em 2017. Modelos de efeitos aleatórios foram desenvolvidos para estudar a relação entre as taxas de mortalidade e as concentrações de PMP. O custo econômico das mortes prematuras foi avaliado usando a abordagem “disposição a pagar” para monetizar as perdas de bem-estar e o método do capital humano para estimar as perdas de produção. As concentrações de PM_10_ estão positivamente associadas à mortalidade por doenças respiratórias e acidente vascular cerebral. Com base em 10.342 mortes prematuras em 2017, as perdas no bem-estar social subiram para EUR 36,227 bilhões (3,1% do PIB espanhol). O valor econômico das perdas de produção presentes e futuras atingiu os EUR 229 milhões (0,02% do PIB). Do ponto de vista social, a poluição do ar é um problema de saúde pública que tem grande impacto na saúde e na qualidade de vida. Os resultados evidenciam a necessidade de implementar ou fortalecer políticas públicas regulatórias, fiscais e de saúde para obter benefícios substanciais à saúde com a redução da exposição.

## Introduction

Air pollution was identified as a leading cause of global disease burden, especially in low- and middle-income countries in 2015 ^1^. The World Health Organization (WHO) estimates that around 4.2 million deaths were attributable to outdoor air pollution in 2016 ^2^. In Europe, estimates suggest that air pollution constitutes both a major cause of premature death and disease and the most important environmental and modifiable health risk ^3^ ^,^ ^4^. A vast body of literature confirms that air pollution significantly impacts the population’s health, with considerable economic effects due to increased premature mortality and morbidity ^5^ ^,^ ^6^.

One of the main objectives of this study is to offer evidence of the adverse effects of air pollution on the population’s health. Among the different types of pollutants, this study focuses on the impacts of particulate matter (PM) exposure. The main reasons for choosing this pollutant are the following: firstly, in comparison with gaseous co-pollutants, clinical studies have shown that PM presents a higher impact on health ^7^. Secondly, regardless of the PM concentration, the literature suggests no safe level of exposure that would avoid affecting people’s health ^8^ ^,^ ^9^ ^,^ ^10^ ^,^ ^11^.

A wide range of epidemiological studies support the assumption that PM exposure negatively affects human health. Mortality rates are often used to study the health impacts on populations subject to PM exposure ^12^ ^,^ ^13^ ^,^ ^14^ ^,^ ^15^ ^,^ ^16^. The effects of PM exposure are well documented for cardiovascular and respiratory system ^13^ ^,^ ^17^ ^,^ ^18^ ^,^ ^19^ ^,^ ^20^ ^,^ ^21^ ^,^ ^22^ ^,^ ^23^ ^,^ ^24^. New evidence also suggests a link between PM exposure, the cerebrovascular system, neurodevelopment, cognitive function, and metabolic diseases such as obesity and diabetes mellitus, which themselves configure risk factors for cardiovascular diseases ^6^ ^,^ ^25^.

In comparison to cardiovascular and respiratory diseases, the evidence linking stroke and PM exposure is less robust, as is the knowledge about the mechanisms underlying this relation. Nevertheless, an increasing number of epidemiological studies have found evidence in favor of the contribution of air pollution to stroke mortality. Maheswaran & Elliott ^26^ found that living near main roads is associated with a significant but small excessive risk of mortality from stroke. Stafoggia et al. ^27^ conducted a multi-country study and reported that overall stroke incidence increased by 19% per 5μg/m^3^ increase in PM_2.5_. Zhang et al. ^28^ found a similar result for PM_10_: stroke mortality increased by 49% per 10μg/m^3^ increases in PM_10_ concentrations. Nevertheless, other studies have found no association between PM exposure and stroke ^29^ ^,^ ^30^.

Estimates for 2010 showed that chronic obstructive pulmonary disease, acute lower respiratory illness, and lung cancer related to PM_2.5_ concentrations, causing around 765,000 premature deaths worldwide ^31^. Medical studies have shown that PM exposure results in pulmonary oxidative stress and inflammation, which is associated with the development of asthma and chronic obstructive pulmonary disease ^7^ ^,^ ^32^ ^,^ ^33^ ^,^ ^34^. 

Among the epidemiological studies on the association between PM exposure and mortality in Spain, Cárdaba et al. ^35^ estimated the burden of mortality from exposure to PM_10_ and PM_2.5_ in the municipality of Valladolid (Spain), pointing out a detrimental effect on mortality from exposure to these pollutants. Alonso et al. ^36^ studied the relation between PM_10_ exposure and mortality (respiratory and cardiovascular diseases and all causes of death) in five Spanish municipalities from 2000 to 2003. In the long term, the number of total attributable deaths per year related to exposure over the 20µg/m^3^ limit amounted to 68 per 100,000 inhabitants.

The European Union (EU) and Spanish public authorities have recently applied regulatory policies that positively affected the task of improving air quality. This is the case of the Clean Air Package, proposed by the EU and adopted in 2013. Statistics have shown a 12% reduction in premature deaths from environmental PM from 2005 to 2010 but these reductions were relatively modest, and the authors believe that the EU should undertake more efforts in this field, including, e.g., reducing limit values for PM to the recommendations of the WHO guidelines ^37^.

The Spanish government has also applied sectoral measures in line with energy and climate change national policies (e.g., reducing emissions associated with coal-fired power generation, encouraging energy efficiency and renewable energy innovations, transport mobility measures, among others). The National Atmospheric Pollution Control Programme 2019-2020 is the latest initiative in this field. Madrid, the capital of Spain, implemented the Air Quality and Climate Change Strategy (approved in 2017). Recent studies show the positive health impacts of reducing PM_2.5_ and NO_2_ regarding deaths from all foreseeable causes due to long-term exposure, with important health benefits related to that regional strategy ^38^. Fiscal instruments have also shown the efficacy of reducing the quantity of pollutants or improving the incorporation of clean technologies, for example, in industries (the same takes place for subsidies to reduce emissions). Nevertheless, Spain has remained relatively distant from the use of these instruments ^39^ ^,^ ^40^. 

This study aims to analyze the relation between PM exposure and mortality rates due to chronic respiratory diseases; tracheal, bronchus, and lung cancer; stroke; and diabetes mellitus from 2001 to 2016 in Spain and to estimate the economic impact of premature death due to PM in Spain according to welfare and production losses in 2017, the most recent year for which data are available. 

## Data and methods

### Health data

The burden attributable to PM was estimated using the criteria specified by the Global Burden of Disease (GBD) risk factors for the following diseases: chronic respiratory diseases; tracheal, bronchus, and lung cancer; stroke; and diabetes mellitus ^1^. The GBD study estimated the burden of diseases attributable to 79 risk factors (including environmental air pollution) in 195 countries and territories from 1990 to 2018. Data is divided by age and sex.

Since a higher risk among exposed populations translates into a higher proportion of deaths attributed to air pollution, the three values of relative risk (upper, base, and lower) were applied to the number of deaths in Spain over the considered period. The data used to estimate premature deaths were obtained from the microdata in the death statistics according to cause of death provided by the Spanish National Statistics Institute ^41^. Deaths are registered following the 10th revision of the International Classification of Diseases (ICD-10). Data were divided by the following codes: J00-J99 (chronic respiratory diseases); C33-C34 (tracheal, bronchus, and lung cancer); I60-I69 (stroke); and E10-E14 (diabetes mellitus). Data provide annual information about the underlying cause of death and victims’ sex, age, and place of residence.

**Figure 1:**
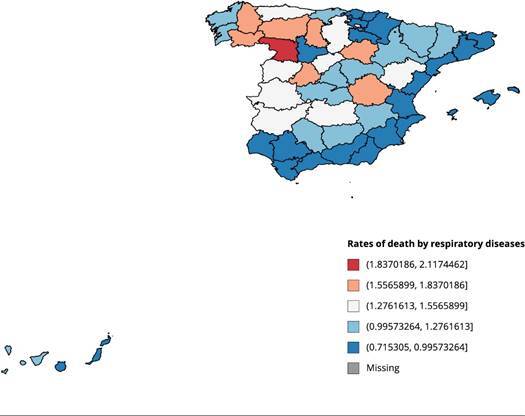
Rates of death by respiratory diseases in the Spanish regions (per 1,000 inhabitants), 2016.

**Figure 2:**
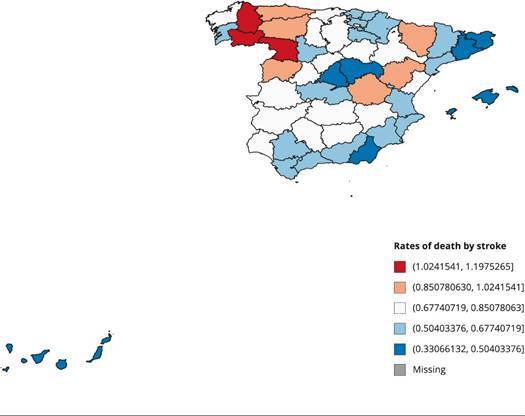
Rates of death by stroke in the Spanish regions (per 1,000 inhabitants), 2016.

Figures 1 and 2 show the death rates by respiratory diseases and stroke in the Spanish regions in 2016, respectively. As can be observed, the eastern-Mediterranean regions show the lowest incidence of deaths from respiratory diseases. This is probably attributable to their drier and warmer Mediterranean climate. As for stroke deaths, some north-south regional patterns are also observed, with a higher incidence in the northern regions of Spain.

### Pollutant data

According to the Spanish Ministry of Ecological Transition and Demographic Challenge, concentrations of PM are based on measurements performed by monitoring stations at fixed sampling points that are classified following two criteria: (a) type of area: urban, suburban, and rural stations; and (b) source of pollutants: traffic-oriented, industrial, and background stations ^42^. 

Covering all the Spanish territory, 600 stations evaluate its air quality. Although the number of monitoring stations and the places in which monitoring stations are located vary among Spanish regions, air quality is measured equally by each station. By region, the mean concentrations of PM_2.5_ and PM_10_ pollutants were obtained using the values reported by all monitoring stations every hour for the 365 days of the year. The information collected covers the period 2001-2016 according to data availability.

Focusing on the pollutants with the strongest evidence of effects on human health (PM_2.5_ and PM_10_), the Spanish territory is divided into several geographical zones. The division into zones considers upper and lower threshold values. This method ensures equivalence in air quality evaluations independently of the considered territorial scope, which could be considered a strength of the data used in this analysis. 

**Figure 3:**
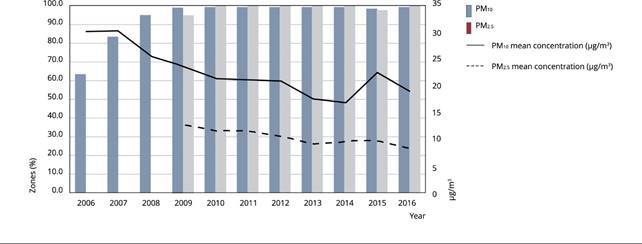
Evolution of the percentage of zones reporting concentrations of particulate matter (PM) below annual limits and annual mean concentrations (μg/m^3^).

The evolution of the percentage of zones in which PM_2.5_ and PM_10_ are equal or below the limits set by the EU Ambient Air Quality Directive is shown in Figure 3. This graph also plots the evolution of the mean concentration of both pollutants over the same years. Regarding PM_2.5_ concentrations, established data are available only from 2009 onward. From 2010 onward, most Spanish regions reported concentrations below the annual limit values of PM_2.5_ and PM_10_. We found a 4.4μg/m^3^ decrease in annual mean concentrations of PM_2.5_ between 2009 and 2016. Spain had always shown high levels of PM_10_ and a significant part of it stems from natural sources, especially African air masses. The evolution in the annual mean concentration for PM_10_ shows a similar decreasing trend, from 30.2μg/m^3^ in 2006 to 18.9μg/m^3^ in 2016. From 2014 to 2015, we found a slight increase in the mean concentration of this pollutant, especially derived from urban zones.

**Figure 4:**
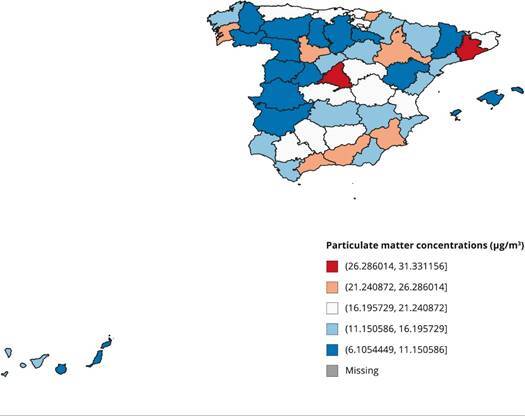
Particulate matter concentrations (μg/m^3^) in the Spanish regions, 2016.

Regional particle concentration in 2016 in Spain is shown in Figure 4. Geographically, a higher concentration of polluting particles can be observed in Madrid and Barcelona, corresponding to the cities with greater development (higher GDP - gross domestic product), population, and other factors (such as the number of registered vehicles). The lowest rates of environmental pollution were found in the western regions bordering Portugal. 

### Control variables

Since our geographical delimitation consists of 50 Spanish regions, different control variables that can influence our outcome of interest were considered. These variables refer to regional development (proxied by GDP), ageing of the local population (proxied by the average age of their inhabitants), and available health resources (proxied by the number of doctors per inhabitant). Competencies in health in Spain are regionally devolved, producing differences in the provision and use of resources between regions. These variables were therefore included in our explanatory model. Data were obtained from the Spanish National Statistics Institute. 

### Econometric approach

To evaluate the relation between mortality rates and PM concentrations, pooled regressions and random-effects models were employed. Pooled regression consists of a standard ordinary least squares (OLS) regression without any cross-sectional or time effects. This kind of estimation is used to derive unbiased and consistent estimates of parameters even in the presence of time-constant attributes. This analysis combines cross-sectional data on the 50 Spanish regions over 16 years to determine its final model. Moreover, the Breusch-Pagan Lagrange multiplier test (LM) for random-effects was applied rejecting OLS regression. The general model has the following basic functional form:

$$y_{it}=\alpha_{i}+x{’}_{it\;}\beta +u_{i}+e_{it}$$

in which *i* and *t* represent region and years, respectively; *y*_*it*_ , the mortality rate for a specific cause of death; *x’*_*it*_ , a vector encompassing the explanatory variables; *α*, individual specific random effects; *u*_*i*_ , a group-specific random element; and *e*_*it*_ , a residual error. PM_2.5_ and PM_10_ concentrations (measured in μg/m^3^) are the variables of interest. A time trend is also included as a dummy variable to control for changes that affect our sample over time (for example, climate effects). Regression models with unbalanced two-way error component disturbances were used to avoid the missing data on pollutants affecting the robustness of our estimations. 

Health data times series were complete for all periods but some missing values were missing for pollution data. Nevertheless, missing data failed to affect the robustness of our estimates since regression models with unbalanced two-way error component disturbances were used. Thus, our matrix gives variable structures for the incomplete data model with random effects.

### Cost estimation methods

Considering the health impacts of air pollution exposure described in the introduction, the economic impacts of premature deaths were assessed by two different perspectives: the Human Capital (*HC*) approach to assess losses in labor productivity and the Willingness to Pay Approach (*WTP*) to evaluate the economic costs of premature mortality according to wellbeing ^43^ ^,^ ^44^ ^,^ ^45^.

The *WTP* approach has become the standard method in high-income countries for evaluating mortality risks associated with air pollution ^46^ ^,^ ^47^ ^,^ ^48^. The approach is based on estimating mortality risks attributable to pollutant exposure, estimated based on the relative risks of death from such exposures and the prevalence of exposure in the populations under study ^46^. The Value of a Statistical Life (*VSL*) includes the full economic costs of premature mortality, consisting of two parts: the first of which (and the most important) refers to intangible losses and the other, to the consumption lost to premature deaths (material losses) ^43^.

The *VSL* concept is equivalent to the rate at which individuals are willing to exchange income and the risk of death:

$$ΜRSi=\frac{\Delta WTP}{\Delta P}$$

in which *MRSI* indicates the marginal rate of substitution between income (reduced by the amount *ΔWTP*) and the risk of death (reduced by the amount *ΔP*) of individual *i*. The average of individual *MRS* provides the *VSL*:

$$VSL=\frac{\Sigma_{i\;}ΜRSi}{n}$$

The specific *VSL* used in this study was estimated by a benefit-transfer approach that assumed a *VSL* base value of USD 3.615 million (2005 prices) ^11^ for the EU27. To estimate the *VSL* for Spain, the following expression was used:

$$VSL_{Spain-2007}=VSL_{ΕU27-2005}\cdot {\left(Y_{Spain-2005}/Y_{ΕU27-2005}\right)}^{0.8}\cdot ΡΡ{Ρ}_{2005}\cdot \left(1+\text{\%}\Delta {Ρ}_{2005-2017}\right)\cdot \left(1+\text{\%}\Delta Y_{2005-2017}\right)$$

in which *VSL*_*Spain-2017*_ refers to the *VSL* for Spain in 2017; *VSL*_*EU27*_ , the average base *VSL* estimate in 2005 for the *EU27* countries; *Y*_*Spain*_ , the Spanish real GDP per capita according to purchasing power parity (PPP) in 2005; *Y*_*EU27*_ , the average real GDP per capita at *PPP* in 2005 of the *EU27* countries; *PPP*_*2005*_ , the purchasing power parity-adjusted exchange rate in 2005 (EUR/USD); (*1 + %ΔP*_*2005-2017*_ ), the inflation adjustment with the consumer price index of Spain between 2005 and 2017; and (*1 + %ΔY*_*2005-2017*_ ), the income adjustment with growth in real GDP per capita in Spain from 2005 to 2017. According to an Organisation for Economic Co-operation and Development (OECD) study ^46^, the income elasticity of *VSL* was assumed to equal 0.8 (central value). For estimates, data on prices, income, and *PPP* rates were obtained from the World Bank database ^49^.

Using this formula, the *VSL* for Spain in 2017 was estimated at EUR 3.724 million (2017 prices). A sensitive analysis was then performed using the upper and lower values of relative risk. A reduction in life expectancy results in current and future production losses, which must be entirely incorporated into the year of death (2017 in our case). Different approaches can evaluate losses of production deriving from a premature dead or morbidity. The Theory of Human Capital is the most widely used approach placing a value on productivity costs in literature ^50^ ^,^ ^51^ ^,^ ^52^. 

To simulate current and future flows for lost work-related income due to premature deaths caused by PM pollution, the following steps were followed:

1) Estimate the expected number of fatalities caused by PM pollution for each cause of death. Applying risk mortality rates, the number of deaths by gender, age, and region of residence were estimated ^53^.

2) Quantify loss of production:

(a) A transition matrix for those who die by age intervals and gender were obtained over our time horizon until retirement age (if individuals survived).

(b) The estimated number of individuals in each gender and age interval is multiplied by average earnings, employment, and survival rates in Spanish statistics. The employment rate is obtained by culling data from the *Economically Active Population Survey* ^53^ ^,^ ^54^ ^,^ ^55^. 

(c) Productivity and discount rates are applied to the resulting matrix. We assumed a 1% increase in productivity - annual average growth in labor productivity from 2000 to 2017 in Spain (OECD, 2018 ^56^) - and a discount rate of 3% (Pinto & Sánchez ^57^) to obtain the current value of future incomes. 

(d) The current value of all cumulative gains obtained over our time horizon for each age interval is then aggregated.

## Results

### Regression results

Table 1 provides our descriptive statistics for the set of variables in our regression analysis. Of the chosen causes of death, respiratory diseases (diabetes) show the highest (lowest) average mortality rates over our set of regions and years, followed by stroke. Respecting average PM concentrations, no pollutant exceeded the recommended EU concentration levels.

**Table 1:** Descriptive statistics.

Characteristics	Definition	Observation	Mean	SD	Minimum	Maximum
Dependent variables						
t_cancer	Tracheal, bronchus, and lung cancer deaths per 100,000 inhabitants (ICD-10 codes: C33-C34)	800	0.46	0.08	0.28	0.72
t_diabetes	Diabetes deaths per 100,000 inhabitants (ICD-10 codes: E10-E14)	800	0.26	0.09	0.06	0.66
t_stroke	Stroke deaths per 100,000 inhabitants (ICD-10 codes: I60-I69)	800	0.81	0.25	0.25	1.68
t_resp	Chronic respiratory diseases deaths per 100,000 inhabitants (ICD-10 codes: J00-J99)	800	1.07	0.28	0.46	2.12
Explanatory variables						
gdp_pc	Annual per capita GDP (2016 prices)	800	22,537.20	5,011.80	13,759.6	41,451.00
age	Average age of the population	800	42.24	2.82	35.84	50.10
physicians	Physicians per 1,000 inhabitants	800	4.52	0.87	2.85	7.62
PM_10_	Particulate matter (< 10µM) concentration (μg/m^3^)	615	26.33	10.84	9.18	99.79
PM_2.5_	Particulate matter (< 2.5µM) concentration (μg/m^3^)	191	10.38	3.00	4.92	20.85

GDP: gross domestic product; ICD-10: 10th revision of the International Classification of Diseases; PM: particulate matter; SD: standard deviation.

Sources: mortality rates were calculated using death statistics according to cause of death ^40^ and the Municipal Register of Population ^62^; GDP per capita has been taken from the Spanish Regional Accounts ^63^; average age came from the Population Structure Indicators ^64^; rate of physicians was constructed using Affiliated Health Professionals Statistics ^65^ and the Municipal Register of Population ^62^; PM pollutants were culled from the Air Quality Database ^41^.

Note: the full sample with 50 regions and 16 years includes 800 observations.

Table 2 shows the results for our regressions on regional mortality rates due to the chosen diseases. As regressions show, mortality rates positively relate only to PM_10_ concentrations, which are statistically significant for respiratory diseases and stroke (random-effects models). However, we found no significance for deaths due to stroke in our random effects estimates, which prohibits us to establish the consistency of the relation between variables. No studied pollutants show statistically significant relations with mortality due to diabetes and tracheal, bronchus, or lung cancer. Pir interpretation of their coefficients shows a small effect. Thus, if PM_10_ concentrations increase by 10%, mortality rates due to respiratory diseases and stroke increase by 0.004 and 0.01 (per 100,000 inhabitants), respectively. R^2^ was highly relevant in the six goodness-of-fit models we developed, showing lower values in diabetes models.

**Table 2:** Pooled and random-effect regressions.

Disease/Pollutant	Pooled OLS				Random effects				LM test
	Coefficients	Robust SE	N	R^2^	Coefficients	Robust SE	N	R^2^	
Respiratory diseases									
PM_10_	0.1123 *	0.0374	615	0.683	0.092 *	0.024	615	Within = 0.132	Probability > χ^2^ = 0.000
								Between = 0.774	
								Overall = 0.663	
PM_2.5_	0.1524	0.093	191	0.678	0.066	0.038	191	Within = 0.329	Probability > χ^2^ = 0.000
								Between = 0.734	
								Overall = 0.648	
Stroke									
PM_10_	0.208 *	0.0422	615	0.471	0.145 *	0.042	615	Within = 0.413	Probability > χ^2^ = 0.000
								Between = 0.003	
								Overall = 0.044	
PM_2.5_	0.1731 *	0.0572	191	0.689	0.024	0.049	191	Within = 0.016	Probability > χ^2^ = 0.000
								Between = 0.389	
								Overall = 0.473	
Diabetes									
PM_10_	0.0332 *	0.0122	615	0.269	-0.01	0.013	615	Within = 0.025	Probability > χ^2^ = 0.000
								Between = 0.27	
								Overall = 0.216	
PM_2.5_	-0.081	0.0595	191	0.16	-0.002	0.036	191	Within = 0.013	Probability > χ^2^ = 0.000
								Between = 0.161	
								Overall = 0.095	
Cancer									
PM_10_	-0.0021	0.0197	615	0.481	-0.01	0.007	615	Within = 0.237	Probability > χ^2^ = 0.000
								Between = 0.516	
								Overall = 0.47	
PM_2.5_	0.0303	0.0218	191	0.688	-0.001	0.016	191	Within = 0.261	Probability > χ^2^ = 0.000
								Between = 0.722	
								Overall = 0.667	

GDP: gross domestic product; LM: Breusch-Pagan Lagrange multiplier test; OLS: ordinary least squares; PM: particulate matter; SE: standard error.

Notes: models include as control variables the logarithm of annual GDP per capita, the average age of population, the number of physicians per 1,000 inhabitants, and a time trend as a dummy variable. PM concentrations are expressed as logarithms.

* Indicates significance at a 1% level.

These results agree with those found in Alonso et al. ^36^, who analyzed the health impact of particulate air pollution in five Spanish cities. Specifically, they found an impact due to exposure to PM_10_ on mortality from respiratory diseases and cardiac causes (0.7/100,000 and 0.4/100,000 people), based on the fraction of mortality attributable to pollution. 

### Economic impact

Table 3 shows the estimated cost of premature deaths due to PM in Spain during 2017, the latest year for which data is available (10,342 fatalities in the base scenario). For this year, the base case shows an estimated cost of EUR 36,226 million (3.1% of Spanish GDP) - an average annual cost of EUR 779 per inhabitant. Mortality due to respiratory diseases accounts for 53.4% of all welfare losses, followed by stroke (16.6%). We established a lower limit at EUR 11,515 million (1% of GDP) and the upper one at EUR 63,057 million (5.4% of GDP).

**Table 3:** Estimating cost of premature death from particulate matter in Spain. Willingness to Pay approach (2017, millions of euros).

Diseases	Relative risks of death		
	Upper	Base	Lower
Respiratory diseases			
Men	17,992.06	10,583.51	3,617.73
Women	14,929.83	8,772.98	2,996.94
Total	32,921.89	19,356.49	6,614.67
Cancer			
Men	7,144.11	4,080.51	1,481.08
Women	2,043.48	1,164.26	434.47
Total	9,187.59	5,244.77	1,915.55
Stroke			
Men	5,315.09	2,639.38	577.71
Women	6,829.42	3,386.86	724.30
Total	12,144.51	6,026.25	1,302.01
Diabetes			
Men	3,915.21	2,484.05	729.00
Women	4,888.39	3,115.21	953.80
Total	8,803.59	5,599.26	1,682.80
Causes of death of the related diseases (%GDP)	63,057.58 (5.4%)	36,226.76 (3.1%)	11,515.03 (1.0%)
Annual cost per capita (EUR)	1,355.1	778.5	247.5

GDP: gross domestic product.

As expected, annual labor income losses from premature mortality are lower than total welfare losses. Table 4 shows the results obtained for labor productivity losses due to premature mortality. The base case (1% annual growth rate of productivity and a 3% annual discount rate) shows an estimated cost of EUR 229 million, equivalent to nearly 0.02% of the Spanish GDP. Production losses related to mortality due to tracheal, bronchus, and lung cancer represent more than 45% of forgone income.

We established a lower limit at EUR 194 million (0.017% GDP), estimating it by considering a 1% increment in productivity and adopting a 5% discount rate. We set an upper limit at 309.8 million euros, following a 0% discount rate (0.027% of GDP).

**Table 4:** Production losses from premature deaths caused by particulate matter in Spain. Human Capital approach (2017, million euros).

Diseases	Discount rates		
	0%	3%	5%
Respiratory diseases			
Men	73,881.04	53,743.72	45,156.56
Women	14,777.13	10,836.57	9,125.73
Total	88,658.16	64,579.29	54,281.30
Cancer			
Men	107,482.55	81,766.73	70,113.18
Women	29,156.83	21,555.03	18,204.95
Total	136,639.38	103,321.76	88,317.13
Stroke			
Men	46,200.63	32,852.29	27,253.49
Women	16,288.10	11,312.83	9,261.03
Total	62,488.73	44,164.12	36,514.52
Diabetes			
Men	18,055.48	13,993.58	12,116.35
Women	3,950.94	3,014.26	2,588.81
Total	22,005.43	17,006.84	14,703.16
Causes of death of the related diseases (%GDP)	309,790.70 (0.027%)	229,071.01 (0.02%)	193,816.10 (0.017%)

## Discussion

Relevant epidemiological studies have shown strong evidence confirming the association between PM exposure and the risk of suffering respiratory and cardiovascular diseases, diabetes, lung cancer, among others. The WHO stated that air pollution configures a major risk for several diseases, leading to disabilities and premature deaths, including heart diseases and stroke as the most common reasons for premature death attributable to air pollution ^25^. Evidence also suggests that the road transport sector now constitutes the leading cause of air pollution-related deaths in Europe ^48^.

This study shows evidence on the health impacts of exposure to PM and estimated the cost of these impacts according to premature deaths. Using mortality data due to those diseases with a high relative risk from air pollution, this study first examined the relation between mortality rates and PM concentrations in 50 Spanish regions. Our models also incorporate other variables the literature recognizes as determinants of health, such as income, population aging, and a proxy of healthcare resource availability. Analyses shows results that agree with previous studies. Mortality due to respiratory diseases and stroke positively relate only to PM_10_ concentrations. Our results show no significant relation between PM and deaths due to diabetes or tracheal, bronchus, and lung cancer.

We assessed the economic cost of premature deaths using two approaches. Based on the WTP approach, our economic estimation was based on 10,342 premature deaths in Spain during 2017, resulting in a cost of around EUR 36,227 million (base case), a figure representing 3.1% of the Spanish GDP. This result hints at the dimensions of its impact if we compare it to labor productivity losses. Using the human capital method, the economic value of current and future production losses by premature deaths reaches EUR 229 million (0.02% of Spanish GDP).

Other studies found similar results. The WHO and OECD estimated the cost to society of PM for the countries in the WHO European Region in 2010 using the WTP methodology ^58^ ^,^ ^59^. Focusing on Spain, this study estimated 14,042 premature deaths due to ambient air pollution. Estimates have suggested that the economic cost of premature deaths for Spain in 2010 total 42,951 million dollars, about 2.8% of its GDP (3.1% in our case). Roy & Braathen ^48^, based on the epidemiological data in the Global Burden of Disease Study, have estimated the incidence of premature deaths due to environmental air pollution and the economic costs of these mortalities.

The World Bank and Institute for Health Metrics and Evaluation (IHME, United States) ^47^ estimate the economic costs of fatal health risks using the same evaluating method (WTP). Their estimates for Spain show that, in 2013, air pollution caused more than USD 49,331 million (2011 prices) in welfare losses, about 3.4% of Spanish GDP (3.1% in our case). Below total welfare losses, annual production losses from premature mortality are estimated to total USD 1,051 million (2011 prices), about 0.01% of its GDP (0.02% in our case).

To our knowledge, this is the first study to combine time with a regional cross-sectional dimension to evaluate the relation between mortality rates and PM concentrations, estimating the economic impact of premature deaths due to PM exposure.

Result extrapolation must consider a series of limitations. Regarding accuracy and availability, no PM concentration data is available before 2009 for the PM_2.5_ pollutant. Moreover, variation in daily air pollutant levels often relates to weather conditions affecting pollutant dispersion and models fail to control these effects. Finally, the World Bank and IHME argue that ground monitoring is insufficient to provide a global coverage to estimate PM exposure that and satellite-based measurements may complement well areas without ground-level monitoring ^47^. The use of indicators from monitoring stations offers an indirect way of measuring the population’s exposure to pollution. This limitation is common to this type of study, which aggregately analyzes the effects of risk factors from pollution impacts on health status. 

We would have liked to evaluate individual-level data to adjust our analyses by other factors (modifiers) that could affect individual mortality. However, this information is unavailable in the microdata from the death statistics according to cause of death (which would offer us homogeneous regional information for the whole country).

Other limitations relate to the methods we used to quantify premature deaths. We would have preferred to use a specific VSL for air pollution in Spain ^55^. Secondly, the main critiques to the Human Capital approach refer to its possible overestimation of production losses. Total production loss will depend on the time companies spend re-establishing initial production level (friction period). Nevertheless, the Friction Cost method is under a great theoretical and empirical controversy because it contradicts some of the axioms in economic theory and the complexity of calculations. Despite these weaknesses, human capital remains one of the most commonly used methods to estimate premature death costs.

From a social perspective, air pollution is a public health concern that greatly impacts health and quality of life. Thus, exposure to air pollution should be a key public health priority for governments. Our results highlight the need to implement or strengthen different types of public policies to reduce air pollution and their effects on individuals’ physical health and wellbeing ^60^ ^,^ ^61^. As an initial consideration and independently of the type of policies public authorities adopt, further quantitative information on the benefits of reducing exposure to these pollutants is needed.
